# Nuclear lamins: Structure and function in mechanobiology

**DOI:** 10.1063/5.0082656

**Published:** 2022-02-01

**Authors:** Amir Vahabikashi, Stephen A. Adam, Ohad Medalia, Robert D. Goldman

**Affiliations:** 1 Department of Cell and Developmental Biology, Feinberg School of Medicine, Northwestern University, Chicago, Illinois 60611, USA; 2 Department of Biochemistry, University of Zurich, Zurich, Switzerland

## Abstract

Nuclear lamins are type V intermediate filament proteins that polymerize into complex filamentous meshworks at the nuclear periphery and in less structured forms throughout the nucleoplasm. Lamins interact with a wide range of nuclear proteins and are involved in numerous nuclear and cellular functions. Within the nucleus, they play roles in chromatin organization and gene regulation, nuclear shape, size, and mechanics, and the organization and anchorage of nuclear pore complexes. At the whole cell level, they are involved in the organization of the cytoskeleton, cell motility, and mechanotransduction. The expression of different lamin isoforms has been associated with developmental progression, differentiation, and tissue-specific functions. Mutations in lamins and their binding proteins result in over 15 distinct human diseases, referred to as laminopathies. The laminopathies include muscular (e.g., Emery–Dreifuss muscular dystrophy and dilated cardiomyopathy), neurological (e.g., microcephaly), and metabolic (e.g., familial partial lipodystrophy) disorders as well as premature aging diseases (e.g., Hutchinson–Gilford Progeria and Werner syndromes). How lamins contribute to the etiology of laminopathies is still unknown. In this review article, we summarize major recent findings on the structure, organization, and multiple functions of lamins in nuclear and more global cellular processes.

## I. STRUCTURE, ASSEMBLY, AND ORGANIZATION OF NUCLEAR LAMIN INTERMEDIATE FILAMENT PROTEINS

**FIG. 1.**
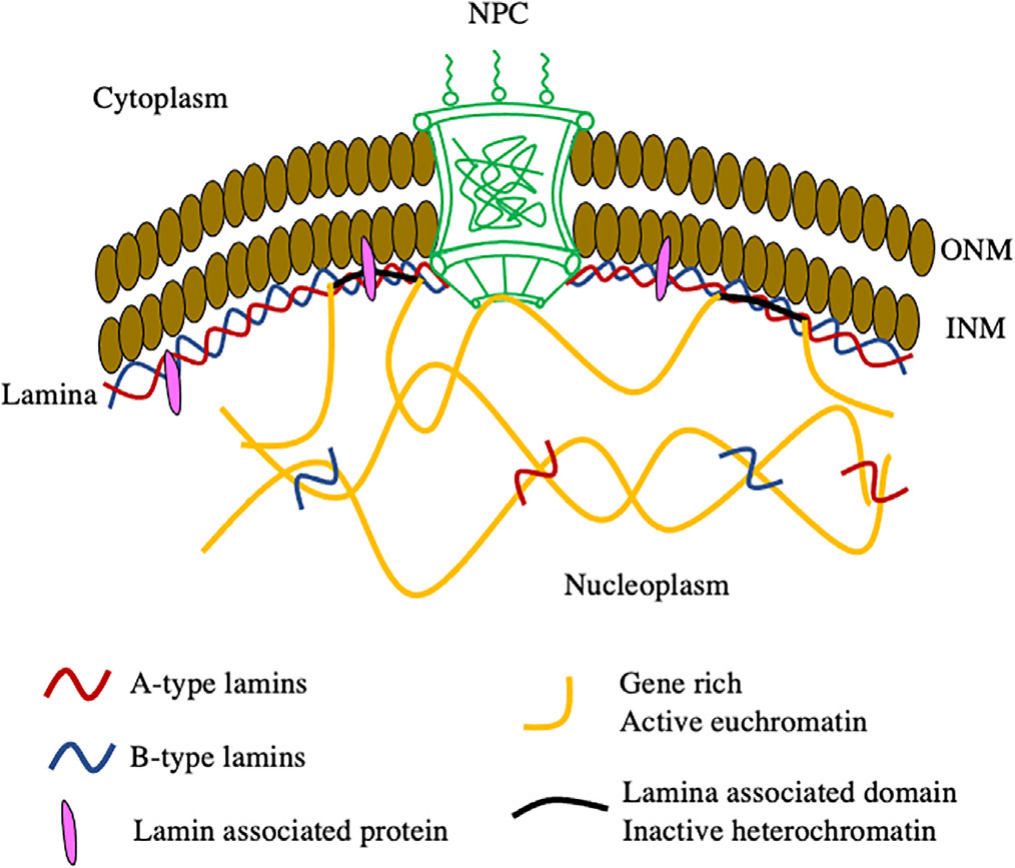
The organization of the nuclear lamins. The INM and ONM seal the NE leaving NPCs as sole openings between the nucleoplasm and cytoplasm. The NL is a meshwork formed by A- and B-type lamins and their associated proteins adjacent to the INM. The A- and B-type lamins at the NL interact with the peripheral heterochromatin lamina associated domains (LADs) and regulate their organization via direct interaction or indirect mechanisms that are mediated by lamin associated proteins. The A- and B-type lamins further mobilize to the nucleoplasm to engage with the active euchromatin domains.

The nuclear lamins are the type V intermediate filament proteins that are major components of the nuclear envelope (NE). The NE is a specialized compartment that physically separates the nucleus from the cytoplasm and provides an interface for linking the genome to the various cytoplasmic cytoskeletal systems and the extracellular environment.^1^ The inner (INM) and outer nuclear membranes (ONMs) form a sealed double membrane structure at the surface of the NE that is permeated by the only known gateways on the nuclear surface, namely, the nuclear pore complexes (NPCs)^2^ (Fig. 1). Juxtaposed to the interior (nucleoplasmic) face of the INM is the nuclear lamina (NL), a ∼10–30 nm thick meshwork of lamin intermediate filaments and their associated proteins (Fig. 1). These lamin filaments act as a nucleoskeletal network that anchors to the INM, NPCs, and peripheral heterochromatin.^3–6^

The lamin family in mammals is subdivided into A-types [lamins A (LA) and C (LC)] and B-types [lamins B1 (LB1) and B2 (LB2)]. The LB1 and LB2 proteins, respectively, encoded by the LMNB1 and LMNB2 genes, are ubiquitously expressed in all mammalian cell types. The LA and LC proteins are alternatively spliced products of the LMNA gene and are expressed in most differentiated cell types.^1^ The A- and B-type lamins primarily localize to the NE in differentiated cells. Additionally, they are also present in the nucleoplasm and play an important role in chromatin organization and gene expression through dynamic binding to both hetero- and euchromatic genomic regions and promoter subdomains.^7–11^ Nuclear lamins are also expressed in other species such as *Caenorhabditis elegans* (*C. elegans*), *Xenopus laevis* (*X. laevis*), and *Drosophila melanogaster*.^12,13^

**FIG. 2.**
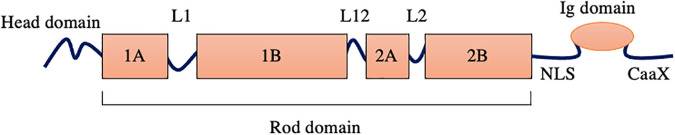
The general structure of lamin intermediate filament proteins. Nuclear lamins consist of the N-terminal (head) domain; the central rod domain, which includes four α-helical domains (coils 1A, 1B, 2A, and 2B) and three flexible linker regions (L1, L12, and L2); and the C-terminal (tail) domain that includes the nuclear localization signal (NLS), the globular immunoglobulin (Ig) fold, and a CaaX motif.

Nuclear lamins are classified as type V intermediate filaments (IF) proteins based on sequence homology.^14^ Like other IF proteins, lamins consist of a central coiled coil (rod) domain composed of four α-helical subdomains (coils 1A, 1B, 2A, 2B) that are separated by flexible linker regions. One difference between lamins and other vertebrate cytoplasmic IF proteins is that lamins have six additional heptad repeats in their central rod domain.^15^ The rod is flanked by an N-terminal (head) domain and a C-terminal (tail) domain containing lamin-specific motifs (Fig. 2). The latter include a nuclear localization signal (NLS), an immunoglobulin (Ig) fold, and a C-terminal CaaX (C, cysteine; a, aliphatic amino acid; X, any amino acid) that is present in lamins A, B1, and B2 but not LC.^16^

**FIG. 3.**
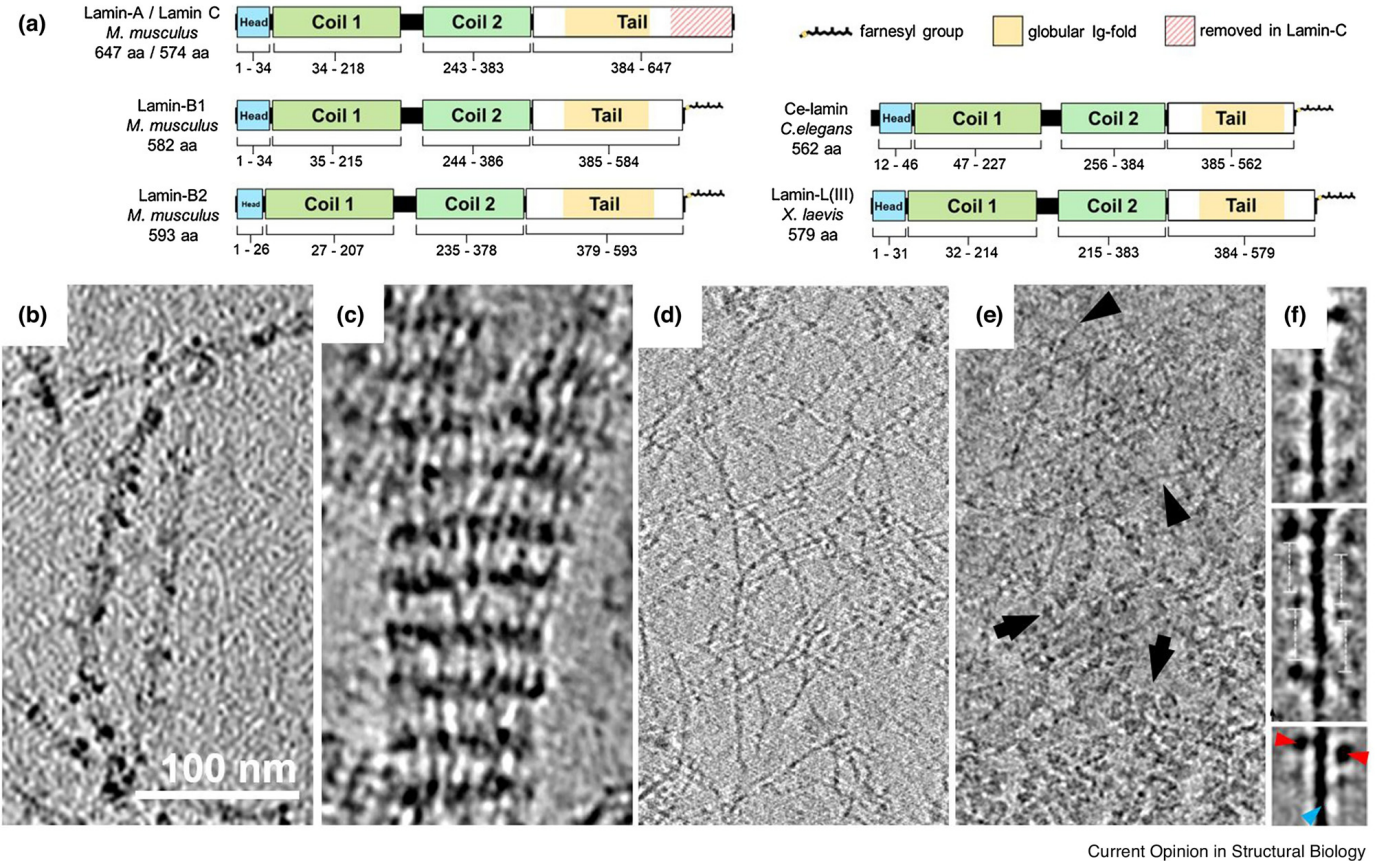
The structure and assembly of nuclear lamins. (a) The structural domains of LA/C, LB1, and LB2 in mammals, Ce-lamin in *C. elegans*, and Lamin-LIII in *X. laevis*. (b) Cryo-ET tomogram of bacterially expressed and purified *C. elegans* demonstrate that lamins assemble into ∼8 nm thick filaments in low ionic strength buffers. (c) Cryo-ET of human lamin A assembled into paracrystaline structures *in vitro*. (d) Lamin filament meshwork is exposed via a cryo-ET slice of a MEF nucleus treated with nuclease. (e) A cryo-ET of native nuclear lamins in MEF nucleus reveals chromatin (arrows) and lamin filaments (arrow heads). (f) 2D averaging shows a 3.5 nm think rod domain (blue arrowhead) with globular Ig-folds (red arrowheads) with a repeat sequence of 20 nm. Reprinted with permission from R. Tenga and O. Medalia, “Structure and unique mechanical aspects of nuclear lamin filaments,” Curr. Opin. Struct. Biol. **64**, 152–159 (2020). Copyright 2020 Elsevier.^2^

Numerous posttranslational modifications (PTMs) occur in lamins. The most extensively studied are in the C-terminus of lamins A, B1, and B2, which possess a C-terminal CaaX motif that is posttranslationally modified in a series of steps beginning with the farnesylation of the cysteine residue.^17^ Following addition of farnesyl to the cysteine of the CAAX motif, the –AAX residues are proteolytically removed from pre-LA by the zinc metalloprotease ZMPSTE24 (CAAX prenyl protease 1 homolog) and from pre-LB1 and pre-LB2 by the endopeptidase Rce1 (CAAX prenyl protease 2). The cysteine is then methylated by isoprenyl carboxymethyltransferase (protein-S-isoprenylcysteine O-methyltransferase) to complete the processing of the CAAX motif. The B-type lamins remain farnesylated for the life of the protein, but the terminal 15 amino acids, including the farnesyl-cysteine, are removed from pre-LA by the protease Zmpste24/FACE1 to produce mature LA. As a result, only B-type lamins remain permanently farnesylated, and thus, their interaction with the INM is retained^16^ [Fig. 3(a)]. Phosphorylation is another major form of PTM in mature lamins, which can regulate lamin solubility^18^ and localization of A-type lamins to the nucleoplasm.^19^

With respect to determining the lamin structure, several systems have employed cell free preparations of purified lamins for *in vitro* assembly assays. Studies of the assembly of mammalian lamins into higher order structures *in vitro* reveal that they do not assemble into individual ∼10 nm diameter IFs seen for most cytoskeletal IF proteins, but rather assemble into paracrystalline arrays^20^ [Fig. 3(c)]. In contrast, Ce-lamin from *C. elegans* can assemble into individual filaments *in vitro* with a diameter of 8 nm [Fig. 3(b)].^21,22^ This difference may be due to the differences in the structure of the lamin rod 2 domain, as the Ce-lamin is shortened by two heptad repeats compared to vertebrate lamins.^23^ Alternatively, it is possible that the filamentous assembly of mammalian lamins is contingent upon specific factors associated with the NE. Thus, it is important to examine the structure of the lamins in their native microenvironment as demonstrated in a recent study using cryo-ET analysis of lamins in mouse embryonic fibroblasts (MEFs).^24^ The results show that mammalian lamins assemble into 3.5 nm thick filaments with an average length of 380 nm. These filaments are the major structural components within a ∼14 nm thick meshwork located immediately subjacent to the INM [Figs. 3(d)–3(f)].^24^ The basic building blocks of these filaments are coiled-coil dimers arranged in parallel and in register.^25^ A fundamental characteristic of lamin filaments is their high degree of flexibility as detected by a persistence length of less than 200 nm.^24^ This short persistence length makes lamins the most flexible (bendable) of all known intracellular “skeletal” filament systems within cells.^26^

Details of the function and precise structural contribution of each lamin isoform to the NL meshwork organization are yet to be revealed, although functional differences and modes of interactions are likely to distinguish the individual lamin types. Using three-dimensional structured illumination microscopy (3D-SIM) and direct stochastic optical reconstruction microscopy (dSTORM) in mouse fibroblasts, it has been shown that each lamin isoform (LA, LC, LB1, and LB2) assembles into a distinct meshwork within the NL.^27,28^ These findings have been confirmed by cryo-ET employing immunogold-labeling^24^ and STORM studies, which also reveal that LB1 and LA/C form spatially distinguishable networks at the nuclear periphery.^29^ Interestingly, the loss of one lamin isoform can impact the structural organization of the other isoform meshworks, indicating that the lamin isoforms interact with each other in the NL. For example, the loss of either LA/C or LB1 in MEFs substantially changes the structure of meshworks in the remaining lamins, whereas the loss of LB2 has a minimal impact on the structure of the other meshworks.^27^ The mechanisms responsible for these interactions remain unknown.

## II. LAMINS AND THE ORGANIZATION OF CHROMATIN

The nonrandom organization of the genome within the nucleus is essential for the regulation of gene expression and repression. In general, gene-rich, transcriptionally active euchromatin is located more toward the center of the nucleus, whereas most gene-poor, transcriptionally repressed heterochromatin is localized adjacent to the NL (Fig. 1).^6^ These latter regions contain lamina associated domains (LADs), which are associated with the nuclear lamins and with other proteins composing the NL^6^ (Fig. 1). The LADs are rich in repressive histone modifications like H3K9me2, H3K9me3, and H3K27me3 and are generally devoid of active chromatin markers such as H3K4me.^30,31^

The contribution of each lamin isoform to LAD organization is an area of active investigation. DNA adenine methyltransferase identification (DamID) maps for LB1, LB2, and LA are very similar genome wide suggesting that each lamin may interact with the same LAD but with minor variations in their frequency.^32^ However, Chromatin immunoprecipitation followed by sequencing (Chip-seq) analysis of micrococcal nuclease-digested chromatin from HeLa cells reveals particular LAD regions that are unique to LA/C or LB1.^33^ Depletion of all lamins in *Drosophila*^34^ or mammals^35^ alters the state of chromatin organization and affects gene activation or repression patterns. In *Drosophila*, depletion of the single B-type lamin results in detachment of many genes from the NL.^36^ Similarly, studies in mammalian cells have shown that depletion of A-type lamins in differentiated cells is sufficient to disrupt LAD organization despite the presence of B-type lamins.^37,38^ These findings suggest that both A-type and B-type lamins are likely involved in organizing LADs. However, it remains unclear whether lamins directly mediate LAD organization or whether their disruption displaces lamina associated proteins that in turn organize LADs.

In addition to their presence at the NL, A- and B-type lamins also localize to the nucleoplasm (Fig. 1).^8–10^ Fluorescence correlation spectroscopy studies demonstrate that A- and B-type lamins form separate, but interacting, nucleoplasmic structures; with nucleoplasmic A-type lamins being more dynamic than B-type lamins.^8^ The A-type lamins have been shown to bind both heterochromatic and euchromatic regions,^39^ thereby restricting the mobility of chromatin within the nucleus.^40^ This is supported by studies on the progeria-linked dominant negative mutation of LA, in which depletion of A-type lamins from the nucleoplasm causes significant global disorganization of the heterochromatin markers and de-repression of some genome regions.^41,42^ More recent studies suggest a similar function for nucleoplasmic B-type lamins in gene regulation by showing that lamin B1 also has a crucial role in the 3D organization of the mouse genome during the epithelial to mesenchymal transition.^11^ Overall, these studies suggest a central role for A- and B-type lamins in chromatin organization and gene expression.

## III. LAMINS ENGAGE WITH LINC COMPLEXES TO CONNECT THE NUCLEUS TO THE CYTOSKELETON

**FIG. 4.**
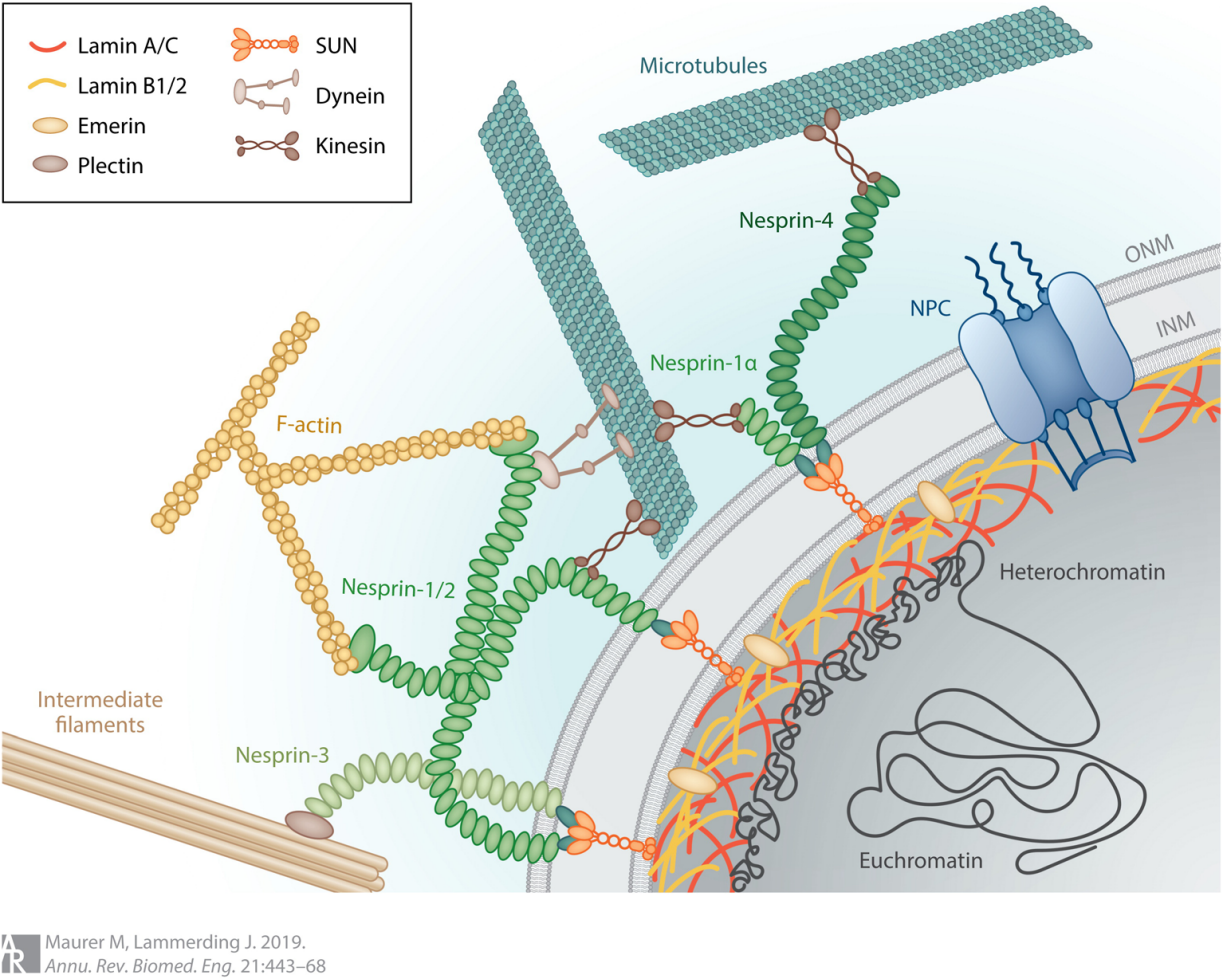
Nuclear lamins facilitate nucleocytoskeletal connections. The LINC complex spans the NE through interplay between its INM SUN and ONM nesprin domains. The nesprin (nesprin-1, -2, -3, and -4) domains of LINC complexes interact with the F-actin, microtubule, and intermediate filament cytoskeletal systems in the cytoplasm via direct binding or adaptor proteins while the SUN (SUN1 and SUN2) domains engage with the A- and B-type lamins as well as lamin associated proteins. The A- and B-type lamins stabilize the SUN, and by extension, nesprin domains of the LINC complexes and their loss are associated with increased mobility of these proteins at the NE, which in turn disrupts cytoskeletal organization within the cell. Reproduced with permission from M. Maurer and J. Lammerding, “The driving force: Nuclear mechanotransduction in cellular function, fate, and disease,” Annu. Rev. Biomed. Eng. **21**, 443–468 (2019). Copyright 2021 Annual Reviews.^47^

The connection between the cell nucleus and cytoskeleton is facilitated by the linker of the nucleoskeleton and cytoskeleton (LINC) complexes, multicomponent structures that span the nuclear envelope. The primary components of the LINC complexes are SUN (*S*ad1p and *UN*c-84 homology) and KASH (*K*larsicht, *A*NC-1, and *S*yne *h*omology) domain proteins.^43^ In mammalian somatic cells, the SUN domain proteins (SUN1 and SUN2) interact with the NL at the INM and bind KASH domains in the perinuclear space (PNS) (Fig. 4). The KASH domain proteins (nesprin-1, -2, -3, and -4) extend from the PNS toward the cytoplasm where they bind to the F-actin, microtubule, and intermediate filament cytoskeletal systems directly or through adaptor proteins (Fig. 4).^44^ The LINC complexes play a central role in regulating nuclear shape, positioning, and movement.^43,44^ Additionally, together with the cytoskeletal systems, they facilitate transmission of forces and mechanical cues from the extracellular environment to the nucleus, which in turn regulate chromatin organization and gene expression.^45–47^ The interactions between the LINC complexes and cell cytoskeleton are also central to cell migration in both normal, e.g., development or wound healing, and pathological, e.g., cancer metastasis, contexts.^48–51^

Lamin isoforms play a significant role in anchoring the LINC complexes to the nucleus, which is essential for nuclear positioning, mechanotransduction, and perinuclear cytoskeletal organization. There is evidence that SUN proteins interact strongly with LA and, to a weaker extent, with B-type lamins.^52,53^ These findings are supported by fluorescence recovery after photobleaching (FRAP) experiments showing increased mobility of GFP-SUN1 and GFP-SUN2 at the NE of MEFs lacking A-type lamins.^54,55^ However, mice with germline deletion of A-type lamins progress to term despite developing severe phenotypes like growth retardation, muscular dystrophy, and dilated cardiomyopathy.^56^ These findings suggest that A-type lamins may not be the only lamins or mechanism(s) participating in anchoring to LINC complexes. In support of this, it has been shown that there are also preferential interactions between B-type lamins with SUN proteins.^57^ FRAP studies of GFP-SUN1 and GFP-SUN2 in MEFs lacking LB2 support this interaction by showing increased mobility of SUN1 and SUN2 at the NE of these cells.^55^ Interestingly, mice lacking LB1, LB2, or SUN1/2 have comparable developmental defects in neuronal migration and brain development further suggesting that B-type lamins may interact with SUN1/2 to stabilize LINC complexes.^58–60^

The interactions of SUN proteins with specific KASH domain proteins (nesprins) of the LINC complexes facilitate interactions between the lamins and F-actin, microtubules, and intermediate filaments in vertebrates.^61^ Specifically, nesprin-1 giant (nesprin-1G) and nesprin-2 giant (nesprin-2G) directly bind to F-actin; nesprin-1, nesprin-2, and nesprin-4 associate with kinesin and dynein motor proteins to interact with microtubules; and nesprin- 3α interacts with cytoplasmic intermediate filaments via plectin^44,62–64^ (Fig. 4). MEFs devoid of A-type lamins show significant changes in the perinuclear cytoskeleton; in particular, the absence or disorganized distribution of vimentin intermediate filaments (VIFs) in the perinuclear areas,^65,66^ a separation between the microtubule organizing center and the nucleus,^67^ an impaired anchorage of transmembrane actin-associated nuclear (TAN) lines,^68^ and the loss or disruption of the highly contractile perinuclear actin caps found on the dorsal nuclear surface.^69^ Perinuclear VIFs are also perturbed in MEFs devoid of B-type lamins, but there are no significant disruptions of perinuclear F-actin organization in these cells.^55^ Interestingly, disruptions in perinuclear F-actin and VIFs in MEFs that lack A-type lamins correlate with an increase in the mobility of nesprin-2G and nesprin- 3α, respectively.^54,55^ Similarly, the finding of a disrupted VIF distribution in MEFs that lack B-type lamins is consistent with increased nesprin- 3α mobility in the NE of these cells.^55^ Together, these studies suggest that A- and B-type lamin isoforms selectively engage with SUN and KASH domains of the LINC complexes to bind and interact with distinct cytoskeletal systems.

## IV. LAMINS CONTRIBUTE TO NUCLEAR AND WHOLE CELL MECHANICS

### A. Lamins regulate nuclear mechanics

Nuclear lamins are key regulators of nuclear morphology, structure, and mechanics.^16,70^ For example, significant changes in nuclear shape occur upon downregulation of lmn-1 in *C. elegans*,^12^ loss of lamin C in *Drosophila*,^71^ LA/C^56^ or LB1^72^ in MEFs, and LB1 or LB2 in mouse cortical neurons.^59^ Early studies using micropipette aspiration experiments demonstrated that the nucleus behaves like a viscoelastic material with power law rheology.^73–76^ Later, it was suggested that the A-type lamins contribute to nuclear mechanics as a highly viscous fluid that impedes nuclear deformation, while B-type lamins serve as elastic walls at the nuclear periphery trying to restore nuclear shape to its original profile following deformation. These findings suggest that the stoichiometric ratio of A-type to B-type lamins regulates nuclear stiffness.^77^ This is consistent with stiffened nuclei that cause impaired constricted cell migration of neutrophils, hematopoietic cells, and cancer cells with increased levels of A-type lamins;^78–80^ or fragility and frequent rupture of nuclei that are partially depleted or devoid of A-type lamins.^81^ However, more recently, micropipette aspiration experiments showed that both A- and B-type lamins contribute to nuclear elasticity, while nuclear viscosity is primarily controlled by the A-type lamins.^82^ This agrees with findings that show loss of LB1 in U2OS cancer cells and LB1 and LB2 in MEFs increase nuclear fragility^83,84^ and compromise nuclear stiffness.^55^ Similarly, fibroblasts from patients with autosomal dominant leukodystrophy, in which lamin B1 is upregulated because of a duplication in the LMNB1 gene, have significantly stiffer nuclei compared to wildtype (WT) controls.^85^ To address these paradoxical findings, it has been suggested that the stiffness of nuclei with low levels of A-type lamins may be more sensitive to changes in A- to B-type lamin stoichiometry while this may not be the case in nuclei with high levels of A-type lamins.^86,87^

The specific contribution of A- and B-type lamins and the mechanisms through which they contribute to nuclear mechanics are active areas of research focusing on determining the mechanical strength of lamin filaments, the regulation of the state of chromatin and its organization, and the regulation of the perinuclear cytoskeletons.

#### 1. Mechanical strength of lamin filaments

**FIG. 5.**
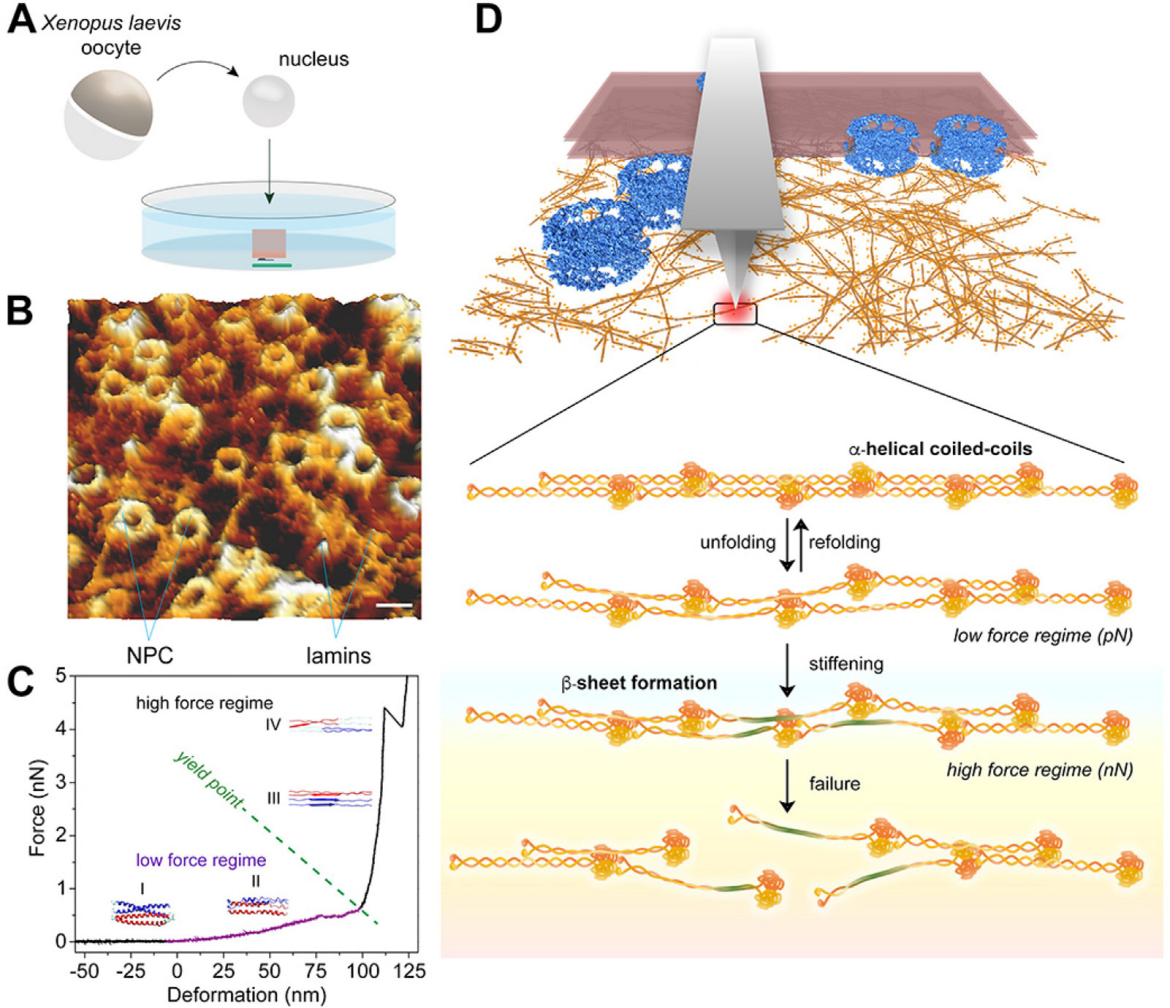
The *in situ* mechanical characterization of lamin filaments. (a) Schematic illustration of the experimental setup for characterization of lamin filaments in nuclei isolated from *X. laevis* oocytes; the nuclei were attached to poly-L-Lysine-coated dishes and then dissected for chromatin digestion and AFM imaging and force spectroscopy experiments. (b) AFM images of the lamina from the nucleoplasmic side showing lamin filaments interconnected with NPCs; scale bar is 100 nm. (c) Typical force-extension curve for nonlinear behavior of a lamin filament. When subjected to mechanical compression, a single lamin filament shows a low force regime with a yield point (the point of transition from a reversible elastic deformation to an irreversible plastic one) from which it undergoes a steep transition to a high force regime followed by failure of the filament. The different force regimes of the filament are assigned to the changes in the lamin α-helical coiled-coils, in which low force regimes (I and II) represent unfolding of the coiled-coil structure, the high force regime (III) denotes the transition from the α-helix to β-sheets, and filament failure (IV) represents the failure of the β-sheets. (d) Schematic model for the lamin filaments response to external forces *in situ*. Reproduced with permission from K. T. Sapra and O. Medalia, “Bend, push, stretch: Remarkable structure and mechanics of single intermediate filaments and meshworks,” Cells **10**, 1960 (2021). Copyright 2021 Author(s), licensed under a Creative Commons Attribution (CC BY) license.

Quantitative rheological studies have shown that reconstituted human LB1 filaments form stiff elastic networks that show significant strain stiffening and resilience (the maximum possible deformation before breakdown) of up to 200% when subjected to shear stresses.^88^ A more recent study used atomic force microscopy (AFM) to examine the mechanics of native single B-type lamin (lamin LBIII) meshworks in *X. laevis* oocyte nuclei (Fig. 5). The large size of frog oocyte nuclei (∼400 μm diameter) and its condensed chromatin structure that does not tightly associate with the lamina allows a direct analysis of *in situ* assembled lamin meshworks by AFM^89^ [Figs. 5(a) and 5(b)]. This study revealed that the lamin meshwork has unique mechanical properties as demonstrated by its reversible deformation at low extension forces (<500 pN) and subsequently the transition to a nonlinear strain stiffening regime at larger strains accompanied by failure at forces greater than 2nN^90^ [Fig. 5(c)]. Assisted by molecular dynamics simulation, the study then suggests that the deformation reversibility in the low force regime is likely due to local unfolding of the α-helical coiled-coils in the lamin filament structure, whereas the strain stiffening of the filaments at higher forces occurs because of transitions in α-helical regions to β-sheet structures [Fig. 5(d)].^90^ This study further demonstrates that lamin filaments can withstand engineering strains as high as 250%, which is comparable to other types of intermediate filaments, e.g., desmin (240%)^91^ and vimentin (205%).^92,93^ By adopting a repetitive force protocol on the lamin filaments and measuring the hysteresis energy, it has also been shown that lamins possess a significant capacity to absorb energy when subjected to smaller or greater compressive forces.^90^ Such capacity confers remarkably high tensile toughness to a lamin filament ( ≈147 MJ m^−3^) that is significantly higher than that of elastin (2 MJ m^−3^), tendon collagen (7.5 MJ m^−3^), or a carbon fiber (25 MJ m^−3^) and is comparable to that of wool (60 MJ m^−3^), nylon (80 MJ m^−3^), and silk (150 MJ m^−3^).^90^ The unique load bearing properties, toughness, and high flexibility of lamin filaments render them central elements of nuclear stiffness and integrity, turning lamins into an optimal material to guard and protect the genome.

#### 2. Lamins and the regulation of chromatin states and organization

Chromatin is a curvilinear 5–24 nm diameter polymer chain with variable 3D concentration distributions at different stages of the cell cycle.^94^ It self-interacts via topologically associated domains^95^ and harnesses LADs to associate with the nuclear lamina at the nuclear periphery.^32^ The structural and organizational properties of chromatin suggest that it may behave like a dynamic crosslinked polymer inside the nucleus that can resist deformation.^86,96–99^ This is supported by studies that show changes in the state of chromatin compaction vs decompaction, or its cross connectivity can alter nuclear stiffness and viscoelasticity.^73,75,76,82,86,96,100–102^ The contribution of chromatin *per se* to nuclear stiffness may be distinct from that of the lamin filaments since chromatin predominantly regulates smaller nuclear deformations (less than 30% strain) while lamins deform during these small deformations and then stiffen to resist larger nuclear deformations.^86,87,103,104^ Nonetheless, as described above (see Fig. 1), lamins interact with heterochromatin at the NL to modulate the organization and state of chromatin. Loss of lamins can result in reorganization of the chromatin and heterochromatin detachment from the NL.^35,37,105^ This suggests that despite their direct contribution to nuclear stiffness, lamins can also indirectly affect nuclear mechanics through their downstream effects on chromatin. For instance, tethering of chromatin to the NE is known to contribute to nuclear stiffness.^106^ Hence, it is likely that the ability of lamins to serve as tethering sites for chromatin/NE binding and, thus, modulating chromatin mobility can have significant effects on nuclear mechanics.^106^ This is consistent with the defective nuclear mechanics in cells from patients with Hutchinson–Gilford Progeria syndrome (HGPS), where the mutant LA protein, progerin, causes defective connections between peripheral heterochromatin and the NL, an overall decrease in heterochromatin throughout the nucleus and softer chromatin.^41,42,107,108^ Similarly, loss of LB1 and LB2 decreases heterochromatin^55,109^ and, consequently, softens the nucleus as measured by small deformation micromanipulations^104^ and AFM measurements.^55^ Furthermore, nucleoplasmic A-type lamins cross link chromatin by directly binding to DNA or through the H2A/H2B core histone proteins and thereby restrict chromatin diffusion and mobility (Fig. 1).^40,110,111^ A recent study found that rescuing LA in mouse embryonic stem cells devoid of all lamin genes (triple knockouts) not only significantly stiffens the nucleus but also increases nuclear viscosity, whereas rescuing LB1 expression stiffens the nucleus but has less of an effect on viscosity.^82^

#### 3. Lamins and the regulation of the perinuclear cytoskeletal distribution and stability

Lamins and chromatin are the dominant intrinsic regulators of nuclear mechanics. However, an emerging body of evidence suggests that the interplay between the nucleus and the perinuclear cytoskeleton can also modulate nuclear mechanics and stability. Computational and experimental studies have shown that changes in cytoskeletal network organization and contractility alter nuclear morphology and stiffness.^112,113^

Microtubules can both stabilize and antagonize nuclear shape and mechanics. In their protagonist role, stabilizing the disrupted perinuclear microtubule network with Paclitaxel in mouse muscle cells mutant for A-type lamins reduces nuclear damage in these cells.^114^ On the other hand, microtubules, along with their associated motors, kinesin and dynein, exert forces on the nuclear envelope that can deform or rupture the nucleus.^106,115–117^ Kinesin/microtubule mediated nuclear movements rather than actomyosin contractions are sufficient to damage nuclei during *in vitro* myofiber differentiation.^114^ Similarly, dynein generated forces on lamin compromised *C. elegans* nuclei enhance the severity of transient NE ruptures and cause NE collapse while lamins counteract these forces on damaged nuclei to allow NE repair.^117^ Additionally, Brillouin microscopy studies show that disassembly of microtubules with nocodazole in NIH 3T3 cells increases nuclear Young's modulus by approximately 33%.^113^

F-actin fibers confine and exert compression forces on the NE, which can deform and rupture the nucleus.^118,119^ Consequently, disruption of F-actin polymerization by latrunculin or cytochalasin D treatment or the inhibition of myosin II by blebbistatin inhibits NE rupture.^118,120,121^ Furthermore, disruption of LINC complexes that facilitate perinuclear F-actin binding to the NE also causes disruption of perinuclear contractile F-actin fibers^44,68,69,119,122^ and attenuates F-actin-induced nuclear compression and rupture.^118^ Therefore, like microtubules, F-actin can also affect nuclear stiffness and protect the nucleus against mechanical deformation. Furthermore, in the absence of A-type lamins, cells cannot form F-actin caps and their nuclei are much more sensitive to stretch induced deformation.^123^ Similarly, in NIH 3T3 cells treated with cytochalasin D, which depolymerizes F-actin, there is a ∼30% reduction in Young's modulus of the nucleus.^113^

Cytoskeletal intermediate filaments are the least studied cytoskeletal system in terms of their contribution to nuclear shape and mechanics. These filament systems are typically concentrated in the perinuclear region, where they form a cage-like or ring-like structure surrounding the nucleus.^63,124,125^ In MEFs expressing VIFs, the importance of this perinuclear cage has been emphasized by demonstrating that it can exert forces on the nucleus and even deform the NE.^124^ Direct force probing of the nucleus using micropipette manipulation has also shown that VIFs can resist nuclear translocation and deformation.^126^ More recent findings demonstrate that a VIF cage protects the nucleus against compressive forces during constricted cell migration,^127^ similar to a previously suggested role for the keratin IFs.^128^ A mechanical link between the lamins and the VIFs in MEFs devoid of A-type lamins or LB2 is supported by a disrupted perinuclear VIF distribution,^55^ a phenotype also present in MEFs with disrupted SUN and KASH domains in the LINC complexes.^125^

### B. Lamins regulate whole cell stiffness and contractile state

Early studies of cell mechanics in lamin-deficient cells found that the cytoplasm in MEFs with reduced levels or devoid of A-type lamins was significantly softer and less viscous compared to WT MEFs.^65,67,129^ Passive microrheology analyses showed that the loss of A-type lamins minimized the normally significant stiffness difference between the perinuclear and lamellar regions in MEFs.^67^ These studies further indicated perturbed interactions between the nucleus and perinuclear F-actin, VIFs, and microtubules^65^ and also showed a separation of the microtubule organizing center from the nuclear surface.^67^ Interestingly, disrupting F-actin networks by latrunculin B or depolymerizing microtubules via nocodazole did not affect the cytoplasmic stiffness in MEFs that lacked A-type lamins, whereas both treatments significantly compromised stiffness in WT MEFs.^67^ These observations suggest that cytoskeletal mediated regulation of the cytoplasmic stiffness is significantly reliant on the structural integrity of the lamin meshworks comprising the NL.

There is also evidence that LA and LC differentially contribute to whole cell mechanics. One study found a strong correlation between the expression levels of lamin C and whole cell stiffness.^130^ Another study found that knockdown of LC in WT MEFs significantly softened the cytoplasm and reduced the cell contractility, while LA knockdown did not soften the cytoplasm but did reduce contractility.^55^ These findings suggest specific functions for LA and LC in modulating whole cell stiffness. Studies on the link between A-type lamin mutations and cell mechanics have also found that a LMNA D192G mutation in cardiomyocytes, which results in severe cardiomyopathy, is associated with attenuated cell adhesiveness.^131^ Furthermore, overexpression of LA in HT1080 fibrosarcoma cells increases the cell stiffness by twofold,^132^ and subjecting these cells to a 5%–15% isotropic stretch attenuates the increase in their spreading area as compared to controls.^133^

Little is known about the role of B-type lamins in cell mechanics. Early indications for such a role came from impaired neuronal migration followed by abnormal brain development in LB1 or LB2 deficient mice.^59,60,134^ Similar studies on heart epicardium development in mice showed that loss of LB1 is accompanied by delays in cell migration, resulting in incomplete development of vascular smooth muscle and compact myocardium at later developmental stages in LB1 deficient embryos.^135^ These findings have led to more in depth studies showing that B-type lamins contribute to cell mechanics and migration.^55^ Nonetheless, unlike the loss of A-type lamins in MEFs that is accompanied by a softer cell cortex and cytoplasm, and reduced contractility, the loss of B-type lamins only softens the cytoplasm and decreases the cell contractility but does not affect the cortex stiffness. These findings further support distinct roles for the A- and B-type lamins in cell mechanics.^55^

## V. LAMINS ARE KEY ELEMENTS IN MECHANOSENSING AND NUCLEAR MECHANOTRANSDUCTION

Early evidence for transmission of force from the extracellular environment to the cell nucleus came from micromanipulation of microbeads attached to the cell surface that showed cytoskeletal reorientation followed by nuclear translocation upon exerting force to the beads.^136^ While transmission of forces to the nucleus can occur independently of nucleocytoskeletal connections,^137–139^ we now understand that a wide range of external forces are transmitted to the nucleus via interaction between the cytoskeleton and the LINC complexes (Fig. 4).^138,140,141^

The application of stresses to the cell surface can instantaneously stretch the chromatin inside the nucleus and upregulate the transcription of a transgene located within the stretched region.^140^ In the same study, knockdown of A- or B-type lamins increased the movement of chromatin, indicating that these lamin subtypes both contribute to the transmission of forces to the nucleoplasm. Consistent with this, lamins can go through posttranslational modifications or conformational changes when the nucleus is under mechanical stress. Studies subjecting isolated nuclei to shear stress have found that there is increased exposure of a cryptic cysteine residue in the Ig-domain if LA/C (Cys522), which is much less accessible in the absence of shear stress.^77^ Increased cellular contractility, and hence cytoskeletal tension on the nucleus, can also cause conformational changes to A-type lamins, lowering the accessibility of specific A-type lamin epitopes at the basal side of the nucleus as compared to the apical surface.^142^ Similarly, an inverse relationship is found between cellular contractility and the phosphorylation state of LA/C, where lower cytoskeletal contractility in cells cultured on soft substrates enhances LA/C phosphorylation, resulting in increased solubility and degradation of the protein and vice versa.^77,143^ Furthermore, direct application of forces to isolated nuclei via nesprin-1 recruits LA/C to the nuclear periphery and stiffens the nucleus.^141^

Lamins may further contribute to nuclear mechanotransduction through their interactions with NPCs (Figs. 1 and 4).^4,144^ The NPCs, which span the NE double membrane, are the major gateways for facilitating exchange of molecules between the nucleoplasm and cytoplasm.^145^ The nucleoplasmic domains of NPCs interact with the NL while their cytoplasmic domains directly associate with the cytoskeleton.^146^ The permeability of NPCs is mechanosensitive.^147,148^ For instance, stretching of the nuclear membrane can dilate the central transport channel of NPCs by about 30 nm, causing more open permeable conformations.^149^ Consistent with this finding, increasing nuclear membrane tension through direct application of forces decreases mechanical restriction to molecular transport across NPCs and promotes translocation of YAP to the nucleus.^148^ Similarly, the release of cellular tension by gentle permeabilization of the cell results in compaction of the NPCs by over 20% accompanied by changes in the nuclear envelope structure.^150^

The mechanosensitive changes to NPC conformations are influenced by the tension of the nuclear membrane and may, in part, stem from the interactions between the NL and NPCs and the mechanical support the NL provides to the nuclear membrane (Fig. 4).^151^ NPCs in MEFs lacking A-type lamins are clustered suggesting an interaction between lamins and NPCs.^28,56^ This is supported by cryo-ET studies, showing a connection between the NL and the NPCs.^24,146^ A super-resolution microscopy study of NPCs in mouse adult fibroblasts that lacked A-type lamins found that the exogenous expression of LA and LC in these cells results in distinct association between NPCs and these lamins.^28^ More recently, another super-resolution study found a strong association between the NPCs and the LA and LB1 meshworks, suggesting a structural link between these lamin isoforms and NPCs.^4^ This study further used immunogold labeling of LA/C and LB1 followed by cryo-ET to examine the contact between the NPCs and lamins. Interestingly, the authors found significantly higher ratios of LA/C over LB1 labeling (6.7:1) in the vicinity of the nucleoplasmic NPC ring, whereas the ratios were much closer in regions without NPCs (1.69:1) suggesting a preference of NPCs for LA/C fibers over LB1.^4^

## VI. SUMMARY AND FUTURE DIRECTIONS

A major function of lamins is their contribution to nuclear and cellular mechanics. Lamin isoforms modulate nuclear stiffness and regulate cellular mechanics and contractility through their distinctive interaction with LINC complexes that bind the nucleus to the cytoskeleton. These roles render lamins as key regulators of nuclear mechanotransduction, which determines how the cellular microenvironment and mechanical cues affect cell behavior and fate. Disruption of lamins, which, in turn, impairs the LINC complex function and intact nucleocytoskeletal coupling, may indeed result in defective mechanotransduction and downstream genomic malfunctions that lead to laminopathies or other disorders such as metastatic cancer. Further in-depth studies are required to elucidate the systematic role of specific lamin isoforms in mechanical and signaling cascades involved in nucleocytoskeletal coupling and transmission of forces to the nucleus. Better understanding of these processes can potentially facilitate development of regenerative approaches and targeted therapies for diseases related to alterations in the structure and function of the lamins.

Lamins are also central elements of the NL and play a major role in nuclear architecture and cellular structure and function. Recent structural studies have revealed that lamin isoforms organize into distinct, but interrelating, meshworks at the nuclear periphery where they interact with the heterochromatin and regulate the genome. Additionally, lamins also localize to the nucleoplasm and interact with euchromatin. Mutations in lamins alter the structure of the NL, affecting its interactions with lamin binding proteins and resulting in altered genome regulation that likely cause laminopathies. Nonetheless, the link between the structure of lamins and their contribution to the etiology of laminopathies is yet to be explored. This is further complicated by the fact that diseases linked to the lamins are tissue specific, suggesting that the structure or function of lamins may be cell-type or tissue specific. Deciphering such fundamental questions requires future studies on how mutations that result in laminopathies affect the structure of nucleoplasmic and peripheral lamins as well as their interplay with their binding partners and chromatin. Recent advances in cryo-ET and super-resolution microscopy along with emerging genomic engineering and analysis techniques could facilitate a better understanding of these principal processes.

## Data Availability

Data sharing is not applicable to this article as no new data were created or analyzed in this study.
